# Health authorities’ health risk communication with the public during pandemics: a rapid scoping review

**DOI:** 10.1186/s12889-021-11468-3

**Published:** 2021-07-15

**Authors:** Siv Hilde Berg, Jane K. O’Hara, Marie Therese Shortt, Henriette Thune, Kolbjørn Kallesten Brønnick, Daniel Adrian Lungu, Jo Røislien, Siri Wiig

**Affiliations:** 1 Centre for Resilience in Healthcare, Faculty of Health Sciences, University of Stavanger, Kjell Arholmsgate 43, 4021 Stavanger, Norway; 2 Faculty of Medicine & Health, School of Healthcare, University of Leeds, Leeds, England; 3 Centre for Age-Related Medicine (SESAM), Helse Stavanger, Stavanger, Norway

**Keywords:** Media communication, Health communication, Risk communication, Pandemic, COVID-19, H1N1

## Abstract

**Background:**

Responses from the H1N1 swine flu pandemic and the recent COVID-19 coronavirus pandemic provide an opportunity for insight into the role of health authorities’ ways of communicating health risk information to the public. We aimed to synthesise the existing evidence regarding different modes of communication used by health authorities in health risk communication with the public during a pandemic.

**Methods:**

We conducted a rapid scoping review. MEDLINE and EMBASE were searched for publications in English from January 2009 through October 2020, covering both the full H1N1 pandemic and the response phase during the COVID-19 pandemic. The search resulted in 1440 records, of which 48 studies met our eligibility criteria**.**

**Results:**

The present review identified studies across a broad interdisciplinary field of health risk communication. The majority focused on the H1N1 pandemic and the COVID-19 pandemic. A content analysis of the studies identified three categories for modes of communication: i) communication channels, ii) source credibility and iii) how the message is communicated. The identified studies on social media focused mainly on content and engagement, while studies on the effect of the use of social media and self-protective behaviour were lacking. Studies on the modes of communication that take the diversity of receivers in the field into account are lacking. A limited number of studies of health authorities’ use of graphic and audio-visual means were identified, yet these did not consider/evaluate creative communication choices.

**Conclusion:**

Experimental studies that investigate the effect of health authorities’ videos and messages on social media platforms and self-protective behaviour are needed. More studies are needed across the fields of health risk communication and media studies, including visual communication, web design, video and digital marketing, at a time when online digital communication is central to reaching the public.

**Supplementary Information:**

The online version contains supplementary material available at 10.1186/s12889-021-11468-3.

## Background

A pandemic “is an epidemic occurring worldwide over a wide area, crossing international boundaries and usually affecting a large number of people…The agent must be able to infect humans, to cause disease in humans and to spread easily from human to human” [[1] p. 2019]. Examples of pandemics are the “Spanish flu” pandemic (1918–1919), the AIDS pandemic (1966-), the “swine flu” pandemic caused by the H1N1 virus (2009–2010), and the recent coronavirus disease 2019, “COVID-19”, caused by the SARS-COV-2 virus (2019-). Pandemic responses are unique in their dependence on expert-based agencies. For instance, pandemic responses during the H1N1 swine flu pandemic were driven by the bureaucratic expert judgement of public health agencies rather than by politicians guided by principles of political survival [2]. Literature reviews of demographic and attitudinal determinants of protective behaviour during pandemic and infectious disease outbreaks have found that participants who see the disease as more severe are more likely to engage in protective behaviour [3–5]. Since pandemics are defined by geography and virology, not by severity, they may cause challenges for risk communication of pandemic infections with low lethality [6]. Furthermore, pandemics spread globally, causing disease in different places at different times. The pathogen can change with time and location and can have effects lasting several years with changing patterns of severity [7], with the potential for devastating impacts on health, economy, and quality of life worldwide [8–11]. Health authorities are challenged by the complexity of pandemic risk communication and the need to reach out to multiple groups of individuals in the public [12, 13].

Risk communication is an interdisciplinary field of research and practice [14]. In the context of public health emergencies, “health communication” is an integrated part of risk communication, which is emphasised by the literature using the term “health risk communication” [13, 15–18]. Health communication and risk communication in public health emergencies, including pandemics, aims to improve health outcomes by influencing, engaging and reaching out to different at-risk audiences with health-related information [12, 19, 20]. Due to the integrative nature of the literature, this review uses the term “health risk communication”. *Trust*, *engagement* and *tailored communication* are among the key concepts in health risk communication, and a lack of these concepts could hinder effective communication [12, 20].

Health risk communication can be transmitted according to different modes of communication. The term *mode* can be described as a way to be or to do things [21], and in the context of this review, it refers to how health authorities communicate risk to the public. New modes of communication and media technology have dramatically influenced health risk communication through the way the public seeks health information online and on social media [22]. However, the rapid transformation in communications technology, including the near-universal use of mobile telephones and the widespread use of digital media, has a major impact on traditional mass media (television, radio and newspapers). Online communication changes how people access and trust health information [12]. Online newspapers and social media had an important role in health risk communication related to the H1N1 pandemic [23, 24] and even more so in the COVID-19 pandemic [25–27]. Social media platforms also offer new possibilities for two-way communication – that is, speaking *with* and not only *to* the public [12, 27, 28]. However, these are not the only modes of communication health authorities use to reach out to the public. Videos, mass media, websites, and prints are examples of other modes of communication that health authorities use in health risk communication with the public.

Previous systematic reviews have synthesised knowledge on risk communication in response to emergent infectious diseases [12, 28] and the H1N1 pandemic [7, 13, 18, 29]. Other reviews have focused on the role of social media during emergent infectious diseases [23, 26] and the COVID-19 pandemic [25, 27, 30] and the role of mass media and public health communication in the COVID-19 pandemic [31]. However, there is currently a lack of synthesised knowledge related to health authorities’ use of diverse modes of communication in pandemic risk communication. Therefore, the aim of this rapid scoping review was to synthesise the evidence regarding the different modes of communication used by health authorities in health risk communication with the public during a pandemic. More specifically, we aimed to obtain a broad overview of the evidence pertaining to diverse modes of communication, irrespective of the study quality, to clarify some key topics and types of outcomes (e.g., knowledge, trust, health literacy, adherence to recommendations) and identify research gaps in correspondence with the scoping review approach [32].

## Methods

The present study applied a rapid scoping review approach that supports a streamlined approach to data identification, extraction, and synthesis [33, 34]. We conducted our review using an adapted version of the Rapid Review approach advocated by the World Health Organization (WHO) [34], guidance for following systematic scoping reviews in healthcare [33], and the Preferred Reporting Items for Systematic reviews and Meta-Analyses extension for Scoping Reviews (PRISMA-ScR) checklist [32]. The WHO defines a rapid review as “…a type of knowledge synthesis in which systematic review processes are accelerated and methods are streamlined to complete the review more quickly than is the case for typical systematic reviews.” [[34] p. 3]. Rapid reviews essentially accelerate or reduce different parts of traditional review methods, which reduces the time taken to move through the process [34]. Scoping reviews are used to obtain a broad overview of the evidence pertaining to a topic and are useful when examining areas that are emerging, such as pandemic risk communication. The scoping review is used to clarify key concepts and identify gaps in line with the review aim [33].

A protocol was drafted and agreed upon with the wider research group prior to commencing the review but not formally registered in line with the rapid review approach [34].

### Eligibility criteria

The “Population-Concept-Context” (PCC) approach was used to specify our rationale and eligibility criteria [32].

#### Concept

We included studies of modes of communication concerning pandemic health risk communication from health authorities to the public. Modes of communication in this paper included but were not limited to web-based information, social media, television, newspapers, video, texts, and narratives. We conceptualised health authorities from a broad perspective, including governments, official health experts, healthcare professionals as official spokespersons, health authority officials, health agencies, and official health bureaucrats, at the regional, national, or international level (i.e., the WHO). We excluded studies concerning health communication between individuals, such as a medical doctor and a patient (e.g., e-health, telemedicine), or between healthcare professionals (e.g., digital educational methods, digital solutions).

#### Context

Pandemics included but were not limited to swine flu (H1N1) and COVID-19. The COVID-19 pandemic is of primary interest, but as it is currently ongoing, searches on this topic could only provide studies on the response phase and use of diverse modes of communication; thus, we included the swine flu pandemic, as it might include knowledge on a long-term perspective on the pandemic. We excluded studies concerning infectious diseases without pandemic potential. Only studies after 2009 were included, which reflects the timeframe of the evidence generated following the last large-scale pandemic (swine flu) and the need for evidence about communication modes to reflect the scale of technological change over the past decade.

#### Population

We included studies of communication to the public and specific target groups in the public without any predetermined categorisation. Commentaries, reviews, opinion pieces, or other papers not reporting primary empirical research were excluded. English-language articles for both qualitative and quantitative peer-reviewed empirical studies were included.

### Search and information sources

We limited our searches to the two bibliographic databases MEDLINE and EMBASE, as advised for rapid reviews [34]. To accelerate the research process and ensure quality through peer review, the search was restricted to peer-reviewed published studies, and no grey literature searches were conducted [34]. In line with the PRISMA guidelines [32], the selection of databases, search terms, and search methodology was determined in collaboration with a university library technician who designed the final search. The final search results were exported to EndNote, and duplicates were removed by a university library technician. After screening pilot searches, the main search was conducted on 28th Oct 2020. We searched using the terms health, risk, mass, crisis, or media communication, communication methods, modes of communication, sources of communication, and H1N1, COVID-19 and/or pandemic. No filters were added in MEDLINE and EMBASE (e.g., language). The searches were limited to 2009-current. The full electronic search strategy for EMBASE and MEDLINE is found in Additional file 1.

### Selection of sources of evidence

**Fig. 1:**
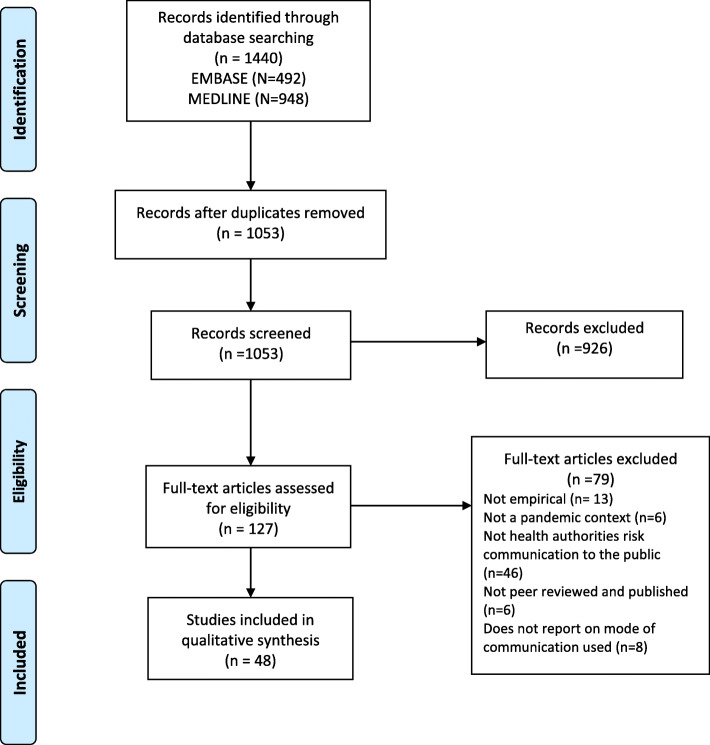
PRISMA flow diagram

The search yielded a total of 1440 hits, of which 492 hits were in EMBASE and 948 were in MEDLINE. Removing duplicates resulted in 1053 unique hits. A total of 127 articles were read in full text and assessed for eligibility, 79 articles were excluded, and 48 articles were included in the review, as displayed in the PRISMA flow diagram (Fig. 1).

As part of the adaptations for undertaking a rapid scoping review [34], one reviewer (SHB) undertook the screening, inclusion, data extraction and charting for included studies, in dialogue with JKOH and SW. SHB screened titles and abstracts using the eligibility criteria outlined above; see additional file 2 for screening questions. A pilot screening was conducted with a pilot search on 16. October 2020 to improve the final search and consistency of the screening process. Full-text screening proceeded against the inclusion criteria to produce a final list of included papers. We did not undertake reference screening or contact with paper authors [34].

### Data charting process and analysis

Following guidance for completing scoping reviews [33], SHB extracted data from included papers in a matrix prior to synthesis: author, year of publication, country of origin, aims/purpose, study population, methodology and sample description, concept, outcomes and key findings relating to the research objectives. Data synthesis was achieved through qualitative content analysis [35]. Pilot testing of the data extraction form was conducted by extracting information from three studies. The pilot-testing was reviewed by JKOH [32]. The results relevant to the review question were summarised, coded, and categorised inductively into three main categories. The analysis was conducted by SHB in collaboration with JKOH, MTS and SW and validated by the co-authors. In keeping with the rapid scoping review approach, we did not undertake an appraisal of the included studies [33]. The main categories described are presented descriptively within each category close to the original findings of the included studies [35]. A table was made to describe the included studies’ reference, context, aim, method and sample, types of outcomes and modes of communication reported (see Table 2). KKB validated the content in Table 2.

## Results

### Study characteristics

**Fig. 2:**
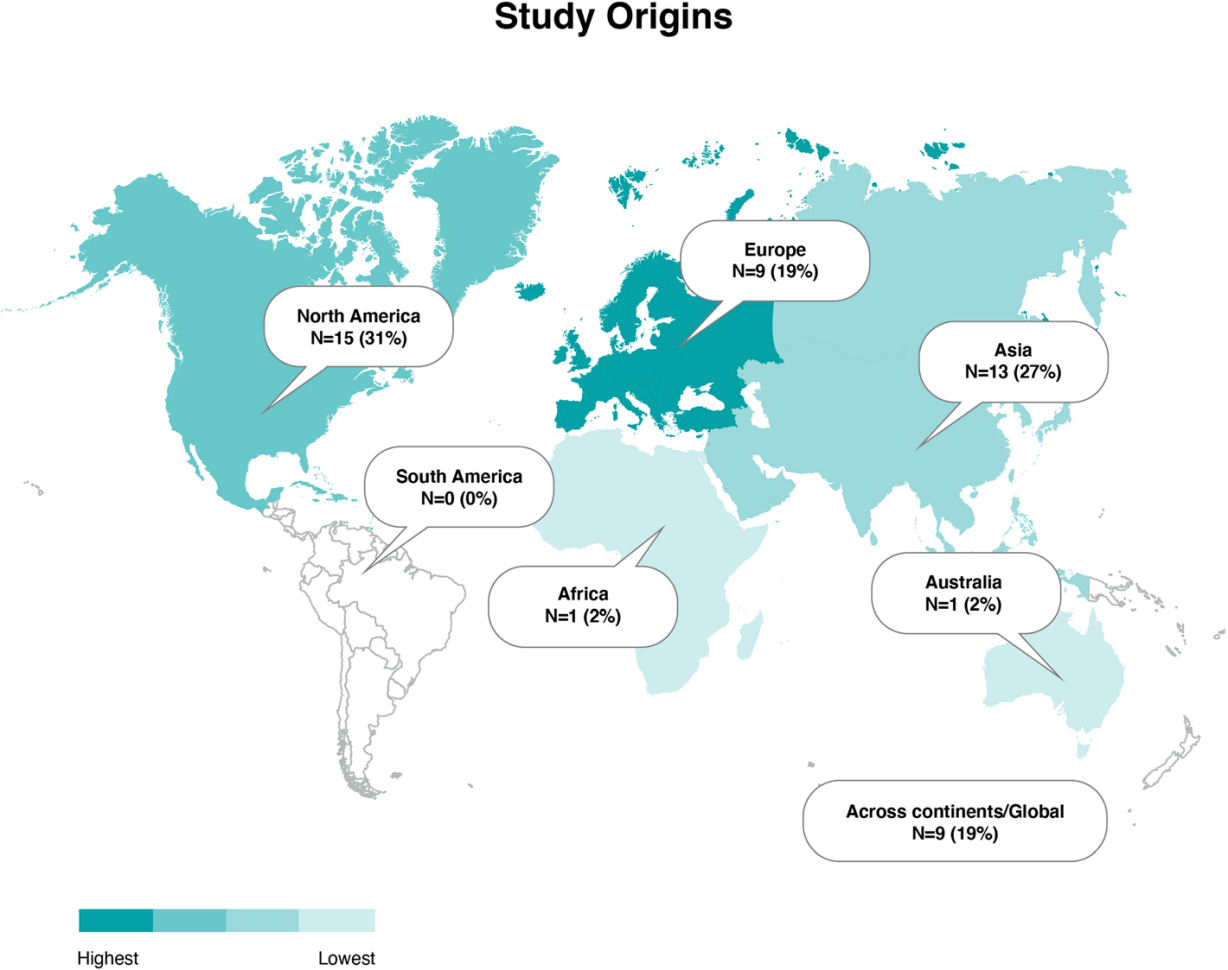
Choropleth of data collection distribution by continents/globally

Of the 48 articles included, 33 included studies concerning the COVID-19 coronavirus pandemic, 12 studies concerning the H1N1 swine flu pandemic, and three studying pandemic influenza in general. The studies’ data collection was conducted in North America (*N* = 15, of which 11 were of US origin), Asia (*N* = 13, of which eight were of Chinese origin), Europe (*N* = 9, of which two were collected in multiple European countries), Africa (*N* = 1), and Australia (*N* = 1). No studies were of South American origin. Nine studies collected data across continents/global studies, of which three studies collected data across continents, five studies collected data on global media use, and one study examined the WHO [60]. The data collection distribution by continents/globally is displayed in Fig. 2.

The methods used were cross-sectional surveys (*N* = 14), one comparative survey [37] and one mixed-method survey and qualitative interview [48]. None of the survey studies had a longitudinal design. Six of the included studies were qualitative interview studies [52, 55, 68, 76, 79, 80]. The study of Kavaliunas et al. [78] was a policy analysis, and the study of King et al. [48] was a case study with multiple qualitative methods. Four studies had an experimental design [73–75, 77], of which the study of Okuhara et al. [75] was a randomised controlled trial (RCT). Eighteen of the included studies applied a quantitative statistical analysis in terms of content analysis or infodemiology studies of diverse media types (television, social media platforms, and YouTube) or webstudies. Three of these studies examined media trends over time (one- to four-month period) [15, 59, 64]. Two studies applied a qualitative content/thematic analysis of media types [54, 56]. The methodological designs in the included studies are displayed in Table 1.

**Table 1:** Methodological design in the included studies

Methods		Count %
**Survey methods** *N* = 15 (31%)	Cross-sectional survey	14 (29%)
	Comparative survey	1 (2%)
**Mixed methods** *N* = 1 (2%)	Survey and qualitative interviews	1 (2%)
**Qualitative methods** *N* = 10 (21%)	Qualitative interviews (individual/focus group)	6 (12%)
	Policy analysis (document analysis)	1 (2%)
	Multiple qualitative methods	1 (2%)
	Qualitative content/thematic analysis	2 (4%)
**Experimental design** *N* = 4 (8%)	Quasi- experimental design	3 (6%)
	RCT	1 (2%)
**Quantitative statistical analysis** *N* = 18 (37%)	Quantitative content analysis	8 (17%)
	Web study	6 (13%
	Infodemiology study with quantitative analysis	4 (8%)

The 48 articles that were included in the review are displayed in Table 2.

**Table 2:** Included studies reporting on health authorities and modes of communications

	Reference	Context	Aim	Method and sample	Types of outcomes	Modes of communication
**Multiple information sources and channels**	Lin et al. [36]	H1N1	To investigate the effect of socioeconomic status and health communication behaviours (including barriers) on people’s knowledge and misconceptions about pandemic influenza A(H1N1) (pH1N1) and adoption of prevention behaviours.	Cross sectional survey with a representative sample (response rate 66%) (*N* = 1569), > 18 years old. USA.	Socioeconomic status, health communication behaviours, knowledge, misconceptions, barriers to information processing. Sources of information.	Multiple information sources. Multiple channels, local television news, national network news, internet, health care professionals, local newspapers, social media.
	Jardine et al. [37]	(SARS) and H1N1	Report on public information use, together with assessed usefulness and credibility, in the province of Alberta, Canada during both the SARS epidemic and H1N1 pandemic.	Comparative survey study. Survey collecting data on the SARS epidemic, representative sample (response rate 47%) (*N* = 1209). Canada. Survey collecting data about the H1N1 pandemic, representative sample (response rate 21%) (*N* = 1206). Canada.	Information sources used (public information seeking), perceptions (usefulness and credibility of information sources).	Multiple information sources, traditional media, social media, friends, doctors, families.
	Al-Hasan et al. [38]	COVID-19	A comparative evaluation of citizens’ adherence process to COVID-19-relevant recommendations by the government.	Cross-sectional survey with a random sample (*N* = 482), 43% USA, 38% Kuwait, 20% South Korea.	Self-adherence to COVID-19 recommendations, information channels reported.	Multiple information sources, newspapers, television, friends, doctors, families and social sources.
	Alanezi et al. [39]	COVID-19	To investigate the situational awareness about COVID-19 in Saudi Arabia and the importance of information sources, information types, and communication channels for creating awareness among the people in Saudi Arabia.	Cross-sectional survey with a non-representative sample (response rate 39%). (*N* = 591), > 18 years old. Saudi Arabia.	Situational awareness (factual knowledge about transmission symptoms and treatment), information sources used, source credibility.	Multiple information sources Online governmental portals and SMS. The Ministry of Health, family and friends as sources of information.
	Ali et al. [40]	COVID-19	This study assessed sociodemographic predictors of the use and trust of different COVID-19 information sources, as well as the association between information sources and knowledge and beliefs about the pandemic.	Cross sectional survey study. Self-selected nonprobability sample (*N* = 11,242), > 18 years old. USA.	Source credibility, information sources reported.	Multiple communication channels. Government websites, television, radio, podcasts, or newspapers.
	Lep et al. [41]	COVID-19	How people search for information, how they perceive its credibility, and how all this relates to their engagement in self-protective behaviours in the crucial period right after the onset of COVID-19.	Cross-sectional survey study. (response rate 43%), (*N* = 1718), 18–81 years old. Slovenia.	Self-protective behaviour and credibility of information sources.	Multiple information sources. Online news portals, television news, social media, official webpage for health risk information, radio. Officials and health care professionals as sources.
	Meier et al. [42]	COVID-19	To describe the public belief in the effectiveness of protective measures, the reported implementation of these measures, and to identify communication channels used to acquire information on COVID-19 in European countries during the early stage of the pandemic.	Cross-sectional survey study. (*N* = 9796), Netherland (*N* = 8611), Germany (*N* = 604), Italy (*N* = 581). 20–70 years old.	Information channels most commonly reported. Accurate information and belief in the effectiveness of protective measures.	Multiple information sources most commonly reported included television newspapers, official health websites, and social media.
	Parsons Leigh et al. [43]	COVID-19	We assessed self-reported public perceptions related to COVID-19 including beliefs (e.g., severity, concerns, health), knowledge (e.g., transmission, information sources), and behaviours (e.g., physical distancing) to understand perspectives in Canada and to inform future public health initiatives.	Cross-sectional survey with a representative sample (*N* = 1996), 18–65 years old. Canada.	Perceptions related to COVID-19, knowledge related to transmission, information sources, and physical distancing behaviour.	Multiple information sources Traditional media sources, print, family, friends, scientific articles, non-government and government and public health websites and social media posts from private sources and from the government.
	Reddy et al. [44]	COVID-19	To assess South Africans’ understanding of and response to COVID-19 during the first week of the country’s lockdown period.	Cross-sectional survey (*N* = 55,823). (≥18 years old). South Africa.	Risk perception, knowledge, trust in information sources, access to information sources, opinions.	Multiple information sources. Government sources, scientific journals, personal doctors, satellite television, radio, local television, print, online news, family, friends, SMS and email.
	Riiser et al. [45]	COVID-19	To describe adolescents’ health information sources and knowledge, health literacy, health protective measures, and health-related quality of life (HRQoL) during the initial phase of the Covid-19 pandemic in Norway. Second, to investigate the association between HL and the knowledge and behaviour relevant for preventing spread of the virus. Third, to explore variables associated with HRQoL in a pandemic environment.	Cross-sectional survey study. (*N* = 2205), 16–19 years old. Norway.	Information sources used. Health literacy (information that is easy to understand).	Multiple information sources, television and family.
	Liao et al. [46]	H1N1	To examine how levels of trust in formal and informal sources of risk/prevention information associated with hand washing and social distancing.	Cross sectional survey study with a representative random sample (response rate 69%), (*N* = 1001), ≥18 years old. Hong Kong.	Source credibility, situational awareness (understanding the cause of H1N1), attitudes, risk perception, reported self-protective behaviour.	Multiple information sources, formal (government/media) information, informal (interpersonal) information.
	Fridman et al. [47]	COVID-19	To investigate associations between public knowledge about COVID-19, adherence to social distancing, and public trust in government information sources and private sources (e.g., FOX and CNN), and social networks to inform future policies related to critical information distribution.	Cross sectional survey study with representative sample (*N* = 1243), ≥18 years old USA.	Source credibility, information sources reported, adherence to social distancing.	Multiple information sources, government sources (webpages), private sources (Twitter, social media, CNN).
	King et al. [48]	H1N1	This study aimed to gain an understanding of parental information seeking, trusted sources and needs in relation to pandemic influenza A 2009 (pH1N1) to inform future policy planning and resource development.	Mixed method study. Survey study (*N* = 431), (response rate 44%). Parents from 16 childcare centres in Sydney. Qualitative in-depth interviews with 42 parents. Sydney.	Information seeking strategies, trusted sources.	Multiple information sources, mass media, hospital and governmental websites, doctors, childcare centres and schools, celebrities, anti-vaccination groups as source of information, mass media, WHO, CDC.
	Liu et al. [49]	COVID-19	This study aimed to clarify the influencing factors for the anxiety level among the Chinese people during the COVID-19 pandemic, with a particular focus on the media exposure to different COVID-19 information.	Cross sectional survey with nonrepresentative sample (*N* = 4991), (response rate 18%), Age 18–61 years old. China.	Risk perception, media exposure, social and geographical proximity to COVID-19.	Multiple information sources. Television, radio, newspaper, interpersonal, weechat, weibo, tiktok, online news website, search engines.
	Gesser-Edelsburg et al. [50]	COVID-19	To examine the response of the Israeli public to the government’s emergency instructions regarding the pandemic in terms of correlations between overall risk perception and crisis management; overall risk perception and economic threat perception; crisis management and compliance with behavioural guidelines; and crisis management and economic threat perception.	Cross sectional survey with nonprobability sampling (*N* = 1056), general public, 18–95 years old). Israel.	Spokesperson’s credibility, trust and health literacy.	Scientific articles, WHO websites, hospital websites.
	Zhang et al. [51]	COVID-19	The objective of this paper is to illustrate the effective process and attention points of risk communication reflecting on the COVID-19 outbreak in Wuhan, China.	Qualitative case study consisting of document analysis and interviews and interviews with governmental officials and experts. China.	Lessons from Wuhan.	Chinese authoritative media and mainstream internet media, social media.
**Mass media**	Hall and Wolf [52]	H1N1	To examine the projected expectations towards the behaviour of the audiences and the projected ways of information circulation informing public health communication strategies during a pandemic.	Qualitative interviews with 31 participants across sectors, including public health agencies. Germany.	Content and framing of messages.	Mass media.
	Rossmann et al. [53]	H1N1	To determine whether media did amplify the A/H1N1- related risks as they were accused of.	A quantitative content analysis of 243 press releases, 1243 quality press and 834 tabloid press articles from WHO, CDC, ECDC, EU Public Health and health ministries in the 10 selected EU countries, between March 2009 and March 2011. Global.	Content of the message, framing of messages.	Newspapers, press releases.
	Basnyat et al. [54]	H1N1	To understand how public health messages provided by the government in Singapore during an Influenza.	Qualitative thematic analysis of 308 government-issued press releases disseminating public health information about H1N1 that was directly linked to news stories (*N* = 56) and news stories about H1N1 generated by the newspaper (*N* = 253). Singapore.	News coverage (framing).	Press releases, newspapers
	Cloes et al. [55].	H1N1	We aimed to assess professional stakeholders’ perceptions of the risk-communication difficulties faced during the 2009 influenza A pandemic in Europe.	Qualitative interviews with 25 experts from 8 European countries were interviewed: 9 from the micro-level, 10 from the meso-level, and 6 from the macro-level of employment.	Trust, perception of risk communication.	Mass media.
	Luth et al. [56]	H1N1	We analyse (1) the content of television news about the H1N1 pandemic and vaccination campaign in Alberta, Canada; (2) the extent to which television news content conveyed key public health agency messages; (3) the extent of discrepancies in audio versus visual content.	Qualitative grounded theory analysis of 47 news clips sampled from the CTV online video archive, and semi-structured interviews with five journalists. Canada.	Content of news, discrepancies in audio versus visual content.	Television news, video and audio content.
**Websites and online platforms**	Khan et al. [57]	COVID-19	To investigate the readability and presence of translated online information readily available to the British public during COVID-19.	Cross sectional web study of google search hits. National Health Service and government websites. Assessed for readability using multiple validated scales. UK.	Readability, health literacy.	Websites.
	Szmuda et al. [58]	COVID-19	To assess the readability of online information regarding the novel coronavirus disease and establish whether they follow the patient educational information reading level recommendations.	Cross sectional web study of google search hits. Websites related to governments, hospitals and health organizations (such as WHO). Assessed for readability using multiple validated scales. Global.	Readability, health literacy.	Websites.
	Lagassé et al. [59]	H1N1	To assess the literacy level and readability of online communications about H1N1/09 influenza issued by the CDC during the first month of outbreak.	Prospective web study. Documents issued by the CDC, USA and Prevention during the first month of outbreak. Assessed for readability using multiple validated scales (i.e., Suitability Assessment of Materials (SAM). USA.	Readability, health literacy.	Websites,
	Fernández-Díaz et al. [60]	COVID-19	To determine whether the content offered to inform about the disease is prepared so that any person can access it, regardless of their technology (hardware, software, or network infrastructure), language, culture, or disability, whether physical or mental, as determined by the Worldwide Web Consortium (W3C).	Web study. Analyses the web accessibility of the WHO website based on guidelines. Six representative pages from the WHO website were selected for in-depth analysis. WHO.	Accessibility of information.	Websites.
	Ringel et al. [61].	H1N1	To assess whether state and local health departments were able to provide online information to their constituents within twenty-four hours of this declaration.	Cross sectional web study. Websites from the health departments of all fifty states, the District of Columbia, USA, and a sample of local U.S. health departments. Assessment of timeliness, how easy it was to find information and the content of the information. USA.	Timeliness (Online information within 24 h).	Websites.
	Hu et al. [62]	COVID-19	To describe and compare the officially released content regarding local epidemic situations as well as analyse the characteristics of information disclosure through local communication in major cities in China.	Cross sectional web study. Analysis of COVID-19 information on official websites of 31 cities. Descriptive statistical analysis. China.	Timely reporting and transparency.	Websites.
**Social media platforms**	Sutton et al. [63]	COVID-19	To examine message retransmission on Twitter, focusing on original messages posted by public agencies responding to COVID-19.	Quantitative analysis of content and structure. Twitter messages from 690 accounts representing U.S. public health, emergency management and elected officials. USA.	Retransmission of messages and engagement (variation in message content and structure).	Twitter, textual messages, and video.
	Sutton et al. [15]	COVID-19	To identify, and describe the patterns of longitudinal risk communication from public health communicating agencies on Twitter during the first 60 days of the response to the COVID-19 pandemic.	Quantitative content analyses of textual content. 138,546 Twitter messages from 696 U.S. Public health agencies’ accounts. USA.	Content of messages.	Twitter.
	Kamiński et al. [64]	COVID-19	To explore the number of reactions to and sentiments of tweets on coronavirus coming from scientific institutions, governmental authorities, and celebrities.	A retrospective infodemiology study. Sentiment analysis. 17,331 COVID-19-related tweets posted by 338 Twitter accounts of health agencies, governmental authorities, universities, scientific journals, medical associations and celebrities in > 4 months since the virus began to spread. Global.	Post impact (number of likes, retweets, and nominal and relative % followers.	Twitter.
	Wang et al. [65].	COVID-19	To investigate the actors’ risk and crisis communication on Twitter regarding message types, communication sufficiency, timeliness, congruence, consistency and coordination.	Quantitative content analysis. 13,598 pandemic-relevant tweets posted over January to April from 67 federal and state-level agencies and stakeholders in USA.	Message types, communication sufficiency, timeliness, congruence, consistency and Coordination.	Twitter.
	Chen et al. [66]	COVID-19	Investigates how Chinese central government agencies used social media to promote citizen engagement during the COVID-19 crisis.	Quantitative content analysis. 1441 Sina Weibo posts. User rating assessment and automated algorithmic text analysis. China.	Emotional valence, engagement, media richness, content type, dialogic loop.	Sina Weibo.
	Liao et al. [67]	COVID-19	To examine public engagement and government responsiveness in the communications about COVID-19 during the early epidemic stage based on an analysis of data from Sina Weibo, a major social media platform in China.	Infodemiology study.Cross-sectional study. Sina Weibo Posts relevant to COVID-19 from Chinese government agency accounts. China.	Public engagement (likes, comments, shares, and followers).	Sina Weibo.
	Ngai et al. [16]	COVID-19	To develop an integrated framework to examine the content, message style, and interactive features of COVID-19-related posts and determine their effects on public engagement in the largest social media network in China.	Infodemiology study. Quantitative content analysis of 608 Sina Weibo posts. China.	Content, message style, and interactive features, engagement.	Sina Weibo.
	Zhang et al. [68].	COVID-19	To illustrate the process of how a piece of information becomes a health rumour. Furthermore, we identify factors that cause people to believe rumours and conduct behaviour that leads to a purchase craze.	Qualitative study. Interviews with 30 participants. Process tracing of media involved in generating misinformation. China.	Perception and behaviour, misinformation.	News report from the authoritative central, official media. Social media.
**Videos**	Li et al. [69]	COVID-19	To evaluate the accuracy, usability and quality of the most widely viewed YouTube videos on COVID-19.	Content analysis of 75 top viewed YouTube videos. Global.	Usability (quality of video content), reliability of videos.	YouTube videos.
	Dutta et al. [70]	COVID-19	To analyse the usefulness of YouTube as a web-based platform for medical and epidemiological information.	Cross-sectional study. YouTube search results analysed for content. 240 videos from non-governmental sources and from government and health agencies. 40 videos in six languages (English, Arabic, Bengali, Dutch, Hindi, and Nigerian Pidgin). Global.	Misinformation.	YouTube videos.
	D’Souza et al. [71]	COVID-19	To assess the most viewed YouTube videos on COVID-19 for medical content.	Coding of video characteristics, source, and medical content. 113 most-widely viewed videos about COVID-19. Global.	Number of views, content of the messages.	YouTube videos.
	Moon and Lee [72]	COVID-19	To compare the reliability, overall quality, title–content consistency, and content coverage of Korean-language YouTube videos on COVID-19, which have been uploaded by different sources.	Infodemiology study. 200 of the most viewed YouTube videos in Korean language from January 1, 2020, to April 30, 2020. South Korea.	Misleading information (usefulness), overall quality, title-content consistency, source, video popularity.	YouTube video.
	Bekalu et al. [73].	Pandemic influenza	To examine if effects of message format vary across audiences of different socio-demographic groups – age, gender, race/ethnicity, education and income.	Experimental study. 627 American adults. Participants were randomly assigned to view either a narrative (*N* = 322) or a non-narrative (*N* = 305) video clip. Pre- and post-viewing questions assessing knowledge and perceived response. USA.	Knowledge, perceived response.	Video.
**Information leaflet**	Krajcovic et al. [17]	H1N1	To analyse the effectiveness of the information leaflet Personal measures during pandemic flu A(H1N1) 2009 with the focus on its design, contents, and distribution	Cross sectional survey. 200 persons in 5 different age groups. Undisclosed response rate. Slovakia.	Reading literacy, comprehensibility.	Information leaflet.
**Graphs**	Banerjee et al. [74]	COVID-19	To investigate the importance of an exponential-growth prediction bias in understanding why the COVID-19 outbreak has exploded.	Quasi-experimental design. Participants from 43 countries. Global.	Bias.	Graphs, numbers.
**Written messages**	Okuhara et al. [75]	COVID-19	To examine the most persuasive message type in terms of narrator difference in encouraging people to stay at home during the COVID-19 pandemic and social lockdown.	RCT. (*N* = 1980). Participants were randomly assigned to five intervention messages (from a governor, a public health expert, a physician, a patient, and a resident of an outbreak area) and a control message. Japan.	Behavioural change (stay at home).	Written messages.
	Mowbray et al. [76]	Pandemic influenza	To examine the persuasiveness of messages promoting vaccination and antiviral use either as health-enhancing or as risk-reducing, as well as messages which conveyed evidence-based information about the costs and benefits of vaccination, or which applied anticipated regret as a motivator for vaccine uptake.	11 focus groups 41 participants from England, including young and older adults, those with lower education, parents, and those with elevated health risk. England.	Persuasiveness, experiences, feelings.	Messages were designed for dissemination through Twitter and social media networks.
	Shulman and Bullock [77]	COVID-19	To address whether the convention to avoid jargon in science communication generalizes to crisis communication as well.	Experimental design with survey. 393 American participants recruited from Mturk Comparing the effects of messages containing jargon (*N* = 197) versus no jargon (*N* = 196) across three topic conditions that vary in situational urgency: COVID-19 (high urgency) flood risk (low urgency, and policy information about how the United States handles national emergencies (control). USA.	Jargon, motivation to process.	Written messages.
**Communication with ethnic minority groups**	Kavaliunas et al. [78]	COVID-19	Our aim is to describe and analyse the Swedish approach in combating the pandemic.	Policy analysis. Data collated from various sources: published scientific studies, pre-print material, agency reports, media communication, public surveys. Sweden.	COVID-19 trends, healthcare system response, policy and measures overview, and implications.	Migrant community leaders to reach out to ethnic groups.
	Driedger et al. [79].	H1N1	How First Nations and Metis people in Manitoba, Canada, responded to the public health management of pandemic H1N1.	Qualitative study. 23 focus groups with 193 people, Aboriginal people in Canada.	Experiences of stigma and trust.	Different formats (radio, television, print, on-line, community sessions).
	Moyce et al. [80]	COVID-19	The purpose of our study was to understand the perception of the Latino community in a rural state regarding COVID-19.	Qualitative study with 14 semi structured interviews with Spanish speaking Latino population in US.	Risk perception and communication needs.	Social media, television, Spanish-language news stations, Facebook.

*Abbreviations*: *CDC* Centers for Disease Control and Prevention, *ECDC* European Centre for Disease Prevention and Control*, WHO* World Health Organization, RCT Randomized Controlled Trial

### Analysis of included studies

The categories describe the evidence related to types of outcomes and the key topics related to how health authorities communicate risk to the public. These were brought together into three main categories: i) communication channels, which describe what media types are used by health authorities, and two additional categories, which describe how and why health authorities use these media types, categorised as ii) source credibility and iii) how the message is communicated.

### Communication channels

Communication channels describe the outcomes related to how people receive pandemic information from health authorities through multiple communication channels, traditional mass media, governmental websites, social media platforms and YouTube videos. Key topics were related to multiple channels: framing, engagement, misinformation, health literacy, self-efficacy, accessibility, and timeliness of the updates.

#### Multiple communication channels

Fourteen of the included studies used survey methodologies with self-reported use of communication channels, with health authorities among the information sources [36–51]. These studies found that people receive pandemic health risk information through a broad spectrum of communication channels and information sources and that they are influenced not only by newspapers, television, printed information, governmental websites, scientific articles, radio, and SMS from the government but also by interpersonal and informal sources, such as friends, family, healthcare professionals, and social media [36–50]. Hence, people are not passive receivers of health risk information but are influenced by the totality of the information they receive from various and multiple communication channels and information sources in addition to health authorities’ health risk communication.

#### Traditional mass media

Five of the included studies of health authorities’ health risk communication and traditional mass media (television and newspapers) reported on outcomes related to framing and the use of mass media as a communication channel [52–56]. A central topic was how framing messages could affect public responses. Framing is defined by Nisbeth [[81], p. 216] as “interpretative story lines that set a specific train of thought in motion, communicating why an issue might be a problem or pose a threat or what might be responsible for it, and what should be done about it”. Studies of media coverage in mass media during the H1N1 pandemic found that the mass media used sensationalist framing of their messages, conflict frames and war metaphors [52–54, 56]. Tabloid papers used risk-amplifying frames when presenting press releases from the WHO and health authorities across 10 European countries during the H1N1 pandemic. Conflict and damage were emphasised when disseminating the press releases [53]. Hall and Wolf [52] interviewed German public health experts and found that the participants attributed fear and panic in the public to sensationalist media coverage.

One qualitative study examining the perceptions of healthcare professionals, regional public health officers, epidemiologists and public health experts across eight European countries following the H1N1 pandemic found that they all experienced collaboration with the media as poor and that the professionals felt misunderstood [55]. The stakeholders emphasised the importance of establishing good relations between national health authorities and the media and highlighted that society’s trust in public health authorities must be improved long before a pandemic [55].

Overall, the evidence describes framing as an important topic in studies of mass media; however, no studies were identified regarding the effect of framing and public responses. Furthermore, no studies reported on outcomes related to health authorities’ communication with the public through radio.

#### Government and health authorities’ websites

Six of the included studies on government websites assessed readability using multiple validated indexes, accessibility, and timeliness of the updates on the websites [57–62]. Cross-sectional Google search studies of the readability of websites found that websites of the UK Health Service government [57] and online educational articles on COVID-19 on websites related to governments, hospitals and health organisations (e.g., WHO) [58] provided information related to COVID-19 that was too difficult for the general population to read. A prospective study of the websites of the U.S. Centers for Disease Control and Prevention during the first months of the H1N1 pandemic classified the documents according to their intended audience and their reading level. The study analysed the material for readability using instruments to assess the suitability of materials, which take into account criteria such as layout, typography, graphics, and surrounding context and its accessibility to an intended audience. The authors concluded that while the webpages were adequately adapted to the reading level of the intended audience, the format and layout (i.e., text-heavy and densely formatted) made the material difficult to comprehend [59]. A study of the accessibility of information on the WHO website found that the information was not accessible for the elderly because the website lacked non-text alternative content for people with vision problems [60]. The lack of timely updated information on webpages has been reported as particularly problematic at the local governmental level in China during the COVID-19 pandemic [62] and during the H1N1 pandemic in the U.S. [61].

The evidence suggests that adaptations for the reading level of the audience alone do not determine information accessibility. The readability, layout, format, accessibility and timeliness of the updates are additional variables that have been studied in assessing the quality of health authorities’ and governmental websites.

#### Social media platforms

Eight of the included studies on the social media platform Twitter and the Chinese microblogging website Sina Weibo reported on message content (i.e., framing), engagement (i.e., message retransmission and number of likes) and misinformation [15, 16, 63–68]. Sutton et al. [63] conducted an analysis of officials’ communication on Twitter and found that retweeting (i.e., reposting or forwarding) of messages is influenced by message content, message features, the organisational type of the account, the number of followers of an account and the time and day the message was sent. Messages were most likely to be retweeted if they contained content related to surveillance, technical information, self-efficacy, or collective efficacy or symptoms. Messages that included video were retransmitted 63% more often than those without video [63]. In a textual analysis of Twitter messages from U.S. public health agencies’ accounts during the first 60 days of the COVID-19 pandemic, Sutton et al. [15] found that the content changed from focusing on instructive messages in the first phase to motivational messages to sustain action in the long term, which focused on the need to protect vulnerable groups.

Five studies explored factors affecting lack of viewer engagement with health authorities’ social media posts related to COVID-19. Kamiński et al. [64] conducted an infodemiology study (an area of science concerning contributed content on the internet) of Twitter messages published by health authorities across the globe. They found that health authorities often used negative framing in Twitter messages on pandemic information compared to celebrities and politicians, who posted optimistic messages that were associated with higher viewer engagement. However, while more positive posts from health authorities may help to enhance engagement, it may also undermine the perceived seriousness of the message. The authors argued for collaboration between health authorities and opinion leaders to succeed in pandemic risk communication [64]. An information dissemination study of U.S. health authorities’ Twitter messages identified inconsistencies and incongruences in messages over time related to critical topics in the communication of COVID-19 information. However, the study did not report on the effect of message inconsistency and people’s self-protective behaviour [65]. Studies of posts on the Sina Weibo platform found low engagement with COVID-19 posts from the Chinese government [16, 66, 67]. The studies suggested that the lack of engagement was related to a failure to use media richness properly [66] and the use of nonpersonal and nonnarrative content [16, 67].

The evidence suggests that health authorities are faced with low engagement with their posts related to pandemic health risk information on social media. Messages containing narratives and a degree of self-efficacy are among the topics that affect engagement on social media platforms Twitter and Sina Weibo. No studies on health authorities’ use of social media in pandemic health risk communication in relation to effects on self-protective behaviour were identified.

#### YouTube videos

Five of the included studies explored the use of the world’s largest video-sharing platform, YouTube [69–73]. Complementing traditional television, online video has become increasingly important. However, videos from credible governmental sources and the WHO are highly underrepresented on YouTube, leaving YouTube vulnerable to the spread of misinformation [69–71]. Li et al. [69] found that 25% of the most popular videos related to COVID-19, which elicited more than 62 million views worldwide, contained misleading content. While governmental videos contained factual information and more accurate COVID-19-specific information, they accounted for only 11% of the videos and 10% of the views [69]. COVID-19-related videos with misleading content were found to have a higher percentage of views on YouTube than those from credible sources [70]. Across nations, the few videos from government and health agencies on COVID-19-related information on YouTube were often found to be credible, but they received a low number of likes and comments [72] and represented a low share of their videos [70]. Two issues that may explain the lack of social media “reach” by health authorities on YouTube are YouTube’s heavy reliance on the English language (videos) and the fact that public agencies tend to have low numbers of subscribers to their YouTube channels [70].

The evidence based on these studies [69–72] suggests that YouTube is a source of misinformation for COVID-19, and health authorities’ videos on YouTube are underrepresented and have low engagement. No studies on health authorities’ use of YouTube videos in pandemic health risk communication that also reported on their effect on self-protective behaviour were identified.

### Source credibility

Source credibility describes key topics related to perceived trust in formal information sources (health authorities, governments, and public health care professionals) related to pandemic health risk information and the impact on self-protective behaviour across media types. Evidence related to governmental approaches to create trust and tailor pandemic health risk communication with ethnic minority groups is described.

#### Trust in formal sources

Ten of the included studies reported data on trust in formal sources, i.e., government sources and health care professionals [37, 41, 46–48, 50, 51, 56, 68, 75]. Trust in formal governmental sources has been associated with more accurate pandemic risk knowledge and self-protective behaviour [46, 47]. In a survey study of Hong Kong adults, trust in formal sources from the government about influenza was associated with greater reported understanding of the causes of H1N1 and more self-reported hand hygiene among males than among those who trusted informal and interpersonal information sources [46]. In a survey study of the US population, trust in governmental sources was positively associated with accurate disease knowledge related to COVID-19. Younger people expressed higher trust in informal sources, such as the CNN news channel and social networks (e.g., Twitter), than US governmental sources, while the older population expressed higher trust in US governmental sources. The authors argue that younger people’s high level of trust in informal sources makes them vulnerable to misinformation and false news [47].

However, credible sources may differ from the sources that are used most often by people. Jardine et al. [37] found that Canadian people used mass media and friends as information sources about the H1N1 pandemic but found public healthcare professionals to be the most credible sources [37]. King et al. [48] found that despite its lack of trustworthiness, the Australian mass media was the most important source of health risk information for parents during the H1N1 pandemic. Medical doctors, authoritative hospitals and government websites, in contrast, were perceived as most trustworthy.

A survey study of a sample of Slovenian adults by Lep et al. [41] found that medical professionals and scientists were perceived as most credible but that news portals, television news and social media were the most used information sources related to COVID-19.

Two qualitative studies reported on failures in governmental risk communication in China [51, 68]. According to Zhang et al. [51], Chinese authorities failed to disclose their uncertainties during the early Wuhan COVID-19 outbreak, which the authors argue undermined their institutional trust. In another qualitative study, Zhang et al. [68] explored the spread of misinformation related to the effect of a Chinese herb on COVID-19 prevention. A message was delivered by one official health expert through the Chinese authoritative media, which affected the perception of the credibility of the message. Social media shared the message using titles inconsistent with the facts, which eventually led to rumours and hoarding behaviour [68].

The evidence describes both positive and negative behavioural outcomes of health risk information from formal sources. However, while the evidence indicates that trust in formal sources is associated with increased knowledge and self-protective behaviour, the studies lacked controls for education level, which may moderate the relationship between gender and age differences and knowledge outcomes. The evidence suggests a disconnect between the information sources people use and find credible and those they report using for pandemic health risk information.

#### Trust in formal spokespersons

Corresponding with the literature review by the WHO on risk communication in public health emergencies [12], five of the included studies found that messages related to pandemic health risk were perceived as most trustworthy when they came from a healthcare professional [37, 41, 48, 56, 75]. An RCT assigned Japanese participants to receive intervention messages from different sources. This study found that health care professionals’ written persuasive messages encouraging people to stay home were more effective than messages from the governor, patients or a public health expert or a control message [75]. Luth et al. [56] conducted a content analysis of television news clips and found that one of the reasons why the Canadian government lost control of its message in mass media was related to the choice of governmental spokespersons in press conferences. When Canadian government officials appeared on television in press conferences with a visual identity as bureaucrats and politicians, they decreased the credibility of their message [56].

Two studies suggest that the credibility of governmental spokespersons and governmental trust are highly related to the evaluation of crisis management [50, 51]. A survey study of public perception of the Israeli government’s emergency management regarding COVID-19 found that the higher the public’s trust and evaluation of crisis management were, the greater the public’s compliance with guidelines. Those who perceived that the prime minister was the most credible spokesperson had a high evaluation of crisis management, whereas those with low evaluation of crisis management trusted other sources of information, such as infectious disease specialists, ministry of health websites and scientific articles [50]. Governmental trust was found to be reduced in China due to the perception that officials had concealed information about the COVID-19 outbreak in Wuhan. This led to reduced government credibility and increased spread of information from unofficial sources, conspiracy theories, and rumours [51]. The evidence suggests that while health care professionals are often perceived as a credible source, trust in governmental sources may not be stable over time. However, no longitudinal studies were identified that studied the variability in governmental trust and media types over time. Although the evidence indicates that trust in healthcare professionals is high, independent of media type, no studies were identified that examined the impact of different media types on people’s trust in formal sources.

#### Tailored communication with ethnic minority groups

Health authorities are advised to collaborate with communities to ensure that their concerns and information needs are understood and to tailor advice and messages to address the target groups [12, 13, 18, 82]. Only three of the included studies reported data on modes of communication and ethnic minority groups [78–80]. One qualitative study with the Latino community in the U.S. found that all respondents wanted more personalised COVID-19 information from researchers or health professionals via communication channels such as personal email, text messaging or a group communication method (e.g., video group call, Facebook group) [80]. A Swedish policy analysis described how the government and decision makers relied on migrant community leaders, representatives of migrant associations, religious leaders, and other influencers to reach out with culturally sensitive information [78]. In a qualitative focus group study of communication priorities for vaccination to indigenous people in Canada, Driedger et al. found [79] that risk messages were transmitted in different dialects and by different formats (radio, television, print, online, community sessions). An additional issue was the need to tailor not only the communication mode but also the message for ethnic minority groups and migrant populations. In a study of indigenous Canadian people, the authors concluded that the communication failed to engender behaviour change because the target group did not understand why they were prioritised for vaccines, instead believing that the government was using them as “guinea pigs” [79].

The evidence from the included studies suggests that ethnic minority groups and migrant populations need personalised information and trusted spokespersons. The review did not identify studies of effective modes of communication with ethnic minority groups.

### How the message is communicated

How the message is communicated reflects the outcomes related to how key topics, including the use of narratives and jargon, emotional valence, and multimodal information, affect health authorities’ pandemic health risk messages.

#### Narratives

According to Slovic [83], people tend to consider their feelings to guide their decision making and judgements, and the use of narratives has been explored in the risk communication literature [14]. Six of the included studies explored how the narrative tone of communications plays a role in the way health authorities’ messages are interpreted and acted upon during pandemics [16, 49, 66, 67, 73, 76]. The choice of narratives has been found to affect uncertainty and anxiety in the public [49], pandemic knowledge [73], engagement [16, 66, 67] and self-protective behaviour (stay home messages) [76]. Liu et al. [49] found that messages negatively affected people’s anxiety when hospitals reported a need for monetary donations, as this reflected a shortage of beds in hospitals. People experienced less anxiety when the government reported opening schools in China, as this provided them with hope [49]. An experimental study found that people reported greater knowledge and greater pandemic influenza prevention measures when they viewed non-narrative health videos about pandemic influenza compared to those who viewed narrative videos [73]. Two infodemiology studies of the Chinese social media platform Sina Weibo found that people were more engaged (more comments and likes) with narrative posts than non-narrative posts, both from the government and from personal sources [16, 67]. Posts related to new evidence and a nonnarrative style were strong negative predictors of the number of shares [16].

A focus group study of English residents found that factual, evidence-based messages related to pandemic influenza from health authorities were most convincing, particularly those that included cost-benefit comparisons of H1N1 vaccination safety. Messages that aimed to elicit feelings of anticipated regret for not getting vaccinated were generally perceived as patronising and unprofessional [76]. Another content analysis of the social media platform Sina Weibo found that posts with high media richness (both text and video) and positive emotions increased citizens’ engagement regarding government social media, while posts high in media richness and negative emotions attenuated citizens’ engagement. Consequently, the authors concluded that plain text should be used when sharing posts that elicit negative emotions, while video should be attached to text when reporting content eliciting positive emotions [66].

The evidence indicates that the use of narratives and messages eliciting emotions in health authorities’ pandemic health risk communication leads to engagement with the public, while people learn more from non-narrative messages than narrative messages. The evidence also suggests that negative narratives may negatively affect people’s emotions.

#### Use of jargon

Four of the included studies reported on outcomes related to the use of jargon in health authorities’ health risk communication [57, 59, 68, 77]. In terms of messages and wording in pandemic health risk communication, the WHO recommends avoiding the use of jargon [12]. However, an experimental study on health messages from health agencies in the context of COVID-19 found no negative effect of jargon on how much difficulty people experience in understanding a message (processing fluency), which may relate to people’s greater motivation to process information that includes jargon when urgent and risky concerns are communicated [77]. Two studies of readability emphasised the importance of adjusting written material to the intended audience. Khan et al. [57] found that the use of jargon and long sentences were contributing factors to the purportedly low readability of UK health authority websites. In a prospective study of the U.S. health authorities’ CDC guidance documents, Legasse` et al. [59] found that while the use of scientific jargon was adjusted to the reading level of different audiences, the documents were still difficult to comprehend due to suboptimal layout (i.e., text-heavy and densely formatted) [59]. The use of jargon in a news report from the Chinese government has also been explored as a contributor to the misinformation and development of rumours in a qualitative study [68].

The sparse evidence based on these studies [57, 59, 68, 77] emphasises that the use of jargon in pandemic health risk messages is multifaceted. Studies report on variables related to the reading level of the audience, misunderstandings, and the recipient’s motivation to process the information.

#### Graphic and audio-visual means

Whether a message is communicated visually through graphics, graphs, moving images, colours, symbols or by the use of text or numbers can have a significant impact on its reach and how the message is received. According to WHO guidelines**,** engaging and effective risk communication is multimodal and includes visual information [12]. Three included studies explored the impact of graphic and audio-visual means [17, 56, 74]. The understanding of exponential growth, for example, which is a key concept during COVID-19, is interpreted differently when communicated through numbers as opposed to visually. A quasi-experimental study with 1980 participants from 43 countries found that people tend to linearise exponential functions when assessing them intuitively by looking at graphs and underestimate future values based on the current value. The study found that showing people prior data in raw numbers before showing them graphs causally reduced exponential-growth prediction bias, which affected risk perception and was associated with improved safety compliance with the WHO’s COVID-19 recommendations [74].

According to Luth et al. [56], public health agencies also need to be aware of the emotional valence and the effect of colours and symbolism in visual media, as they can be used to encourage positive health behaviours or elicit panic. In a content analysis of Canadian television news of the H1N1 pandemic, Luth et al. [56] found the use of static information screens in conjunction with auditive messages that used colours (red) and symbols (technical, alien objects), which may contribute to negative valence [56]. Luth et al. [56] also emphasised the importance of matching news footage with the public health message delivered. Luth et al. [56] found a mismatch between the visual content and the audio content in the presentation of official priority groups for vaccination. Visual footage showed seemingly healthy nonpriority individuals in line to be vaccinated, but the audio targeted vulnerable groups [56].

The perception of colour may also vary depending on the audience and context. A study of an information leaflet used by the Slovak Ministry of Health in communication about the H1N1 pandemic found that the use of white and red types on a dark blue background was preferred by most primary school students. This combination of colours, however, was perceived negatively by more than half of healthcare professionals, presumably because of differences in ageing and different colour perceptions and preferences in children and adults [17]. The authors argue that reading literacy and age are important factors to take into account when tailoring information in health information leaflets [17]. The limited evidence related to graphic and audio-visual means [17, 56, 74] suggests that different modalities affect the message in a multifaceted way. Visual and multimedia messages are interpreted differently than text- or number-based messages. Symbolism, colour perception and preferences change according to audiences and contexts. While the effect and importance of creative and audio-visual production and means is an important area for pandemic health risk communication, no studies of creative communication choices or evaluations/comparisons of various visualisation techniques relating to graphic and audio-visual communication were identified.

## Discussion

### Key topics identified and implications for research and practice

Our review provides novel insights into topics regarding different modes of communication used by health authorities in health risk communication with the public during a pandemic. We found that key topics identified in the health risk communication literature, such as trust, dissemination through multiple channels, framing, narratives, self-efficacy, the use of jargon, health literacy, misinformation, tailored risk communication, multimodal information, and emotional valence, remain relevant to health authorities’ health risk communication during pandemics [12–14, 29].

#### Communication channels

Consistent with other reviews, this review found many studies reporting that people receive information from multiple channels [12, 84]. The studies suggest that people are not passive receivers of health authorities’ information, a wide spectrum of communication channels is used, and meaning making is influenced by both informal and formal sources, as highlighted in the literature [85]. Hence, health authorities are advised to disseminate information through multiple channels, including social media [12, 84]. However, health authorities’ rapid message dissemination on social media suggests inconsistency, which could be a source of confusion for the public [37, 65]. According to Jardine et al. [37], receiving information from multiple sources is only better if the additional information sources contribute to improved informed decision making, less confusion and strengthened credibility. The review findings also emphasise that misinformation is a significant problem across social media sites related to pandemic risk communication. This sometimes results in confusion and even panic for the public [23, 26, 27]. A major challenge for health authorities during pandemics has been to combat the infodemic, the overabundance of information, by identifying and addressing misinformation, rumours, and the contradictory information the public receives from multiple informal sources on social media sites [12, 23, 27, 86]. At the same time, there is a lack of consistency in their own messages that are disseminated rapidly and in parallel on multiple communication channels. As highlighted by Ratzan et al. [20], global health communication demands a communication strategy, particularly on social media, that ensures consistent and congruent messages to the public. Challenges for health authorities across nations are to create engagement in social media, provide the public with webpages that are accessible, updated in a timely way, and tailored towards varying reading levels and with a readable layout to ensure or avoid risk amplification of their messages delivered through mass media.

#### Source credibility

Social trust is “the willingness to rely on those who have the responsibility for making decisions and taking actions related to the management of technology, the environment, medicine, or other realms of public health and safety” [[87], p. 354]. Social trust in health authorities is value-based and relies on judgements of similarities in intentions and values [88]. Corresponding to the existing literature, our review findings suggest that people tend to have social trust in health care professionals as spokespersons and information sources in public health emergencies [12]. Furthermore, the current review indicates that trust in health authorities as an information source is not a static phenomenon; rather, it is highly dynamic and related to the public perception of health authorities’ crisis management [50, 51]. The evidence in the present review cannot conclude why trust in governmental sources varies. In a critical review of the literature, Siegrist [88] found that social trust varies by hazard and respondent groups, and little is known about the heuristics people rely on when evaluating hazards [88]. Factors related to why people choose informal sources of information may relate both to the respondents (e.g., attitudes, education) and to the government’s crisis management or trust in a particular spokesperson. According to Siegrist [88], there is a need to separate trust and confidence. The type of trust described in the present review is related to the concept of “confidence”. Confidence is affected by past experiences of emerging evidence, indicating that events will occur as expected. While the level of confidence in crisis management may affect risk perception and behavioural outcomes, a total loss of social trust is rarely found in response to governmental risk communication (or the lack of such) [88].

The evidence of the current review also corresponds with the literature indicating that health authorities may need to collaborate with trusted spokespersons and tailor communication methods to reach out to immigrant and ethnic populations [12, 13]. Finally, this study corresponds to the knowledge gained from research conducted on epidemics (notably Ebola, Zika and yellow fever), suggesting that efficient modes of communication vary by location and population. What works best for one population might work poorly for another [28]. Hence, health authorities’ health risk communication needs to be adaptive to the pandemic situation and the multiple receivers in the field, be responsive to its development over time, and choose credible spokespersons in media to maintain public confidence in health authorities as a source of health risk information.

#### The content of the message

This review identified topics related to the content of the message, such as the use of narratives, emotional valence, framing and jargon, multimodal messages and providing people with messages that support self-efficacy. Our findings correspond with previous literature findings that health authorities should use balanced and factual information based on science and evidence and should motivate self-efficacy and promote specific actions people can realistically take to protect their health [12, 82, 89]. However, while the WHO guidelines propose that messages should be jargon free and not explained in technical terms to reach the general public [12], we identified one study [77] implying that this may not apply to the COVID-19 context. Existing reviews of the literature have emphasised the impact of education and health literacy on protective behaviour during pandemic and infectious disease outbreaks [3–5] and concluded that individual differences in both attitudes and knowledge about risks suggest that there may be no “one size fits all” approach to risk communication [90]. The use of jargon may be a question of what works for whom.

The evidence to support the choice of message attributes (e.g., narratives, non-narratives, emotional components) in the current review is inconclusive. Although the findings in the present review suggest that people learn more from non-narrative messages, Downs [91] argues that the use of narratives in science communication may help to communicate complex information because it can provide the receiver with a context, capture attention and improve the understanding and processing of the information. According to Balog-Way et al. [14], there is no single dominant formula for risk communication. In a review of the risk communication literature, Balog-Way and colleagues conclude that the effect of single message attributes is typically contingent, indirect and cumulative, and effective risk communication requires a multifaceted approach. Furthermore, it is a matter of what works for whom. The evidence related to jargon and narratives in the current review reflects the complexity health authorities face when designing effective messages for various audiences.

### Research gaps

The research gaps identified in this review are related to health authorities’ pandemic health risk communication and modes of communication. This review was limited to empirical studies of pandemics, which pertained to mainly the H1N1 pandemic and the response phase of COVID-19 (Jan through Oct 2020).

A research gap identified in this review is the lack of high-quality RCT studies to study the effect of modes of pandemic health risk communication on behavioural outcomes. Only four studies with experimental designs were identified in this review. The study by Bekalu et al. [73] has a high risk of bias, as subject flow with attrition was not assessed, and it was not reported whether the analyses were per protocol or intention to treat. The study by Banarjee et al. [74] also lacks a description of recruitment and attrition after starting the experiment; hence, the sample cannot be considered randomised. Furthermore, the quasi-experimental design has a high risk of bias. The study by Okuhara [75] lacks a proper description of the intervention given (persuasive message to stay home) and suffers from a lack of detail on recruitment or attrition and a lack of description of the analytic strategy (intention to treat vs. per protocol). The Shulman and Bullock [77] sample was gathered from “Mechanical Turk”, making recruitment selection very difficult to assess, and these authors did not discuss attrition after randomisation, intention to treat or per protocol analyses. Hence, all the experimental studies had a high risk of bias.

The lack of effectiveness studies and high-quality trials for risk communication is notable in risk and disaster communication research [92]. In a review of the effectiveness of disaster communication, Bradley et al. [92] concluded that it has been difficult to conduct RCTs in risk communication in recent years because people in the intervention and control groups are likely to share information, with a resultant significant likelihood of “contamination”. Additionally, the differences between the studies make it difficult to conclude that one method of risk communication is superior to others [92].

As the rapidly evolving knowledge related to pandemic health risk and the multiple channels used by the public impose challenges for research on health risk communication during pandemics, methodological approaches need to embrace this complexity. Studies with theory-driven approaches to risk management have analysed COVID-19 from a complexity perspective [93–95]. Complex adaptive systems and resilient responses in healthcare have been studied with mixed-method and multimethod case study methodologies, which are relevant to risk communication [96, 97]. Only two case studies of the crisis management/healthcare system were identified [48, 78]. Case studies are needed to gain insight into 1) health authorities’ capacities to respond to the various and complex challenges faced in risk communication through the pandemic and 2) their strategies in using different modes of communication.

Furthermore, three areas of research gaps need to be highlighted. First, we found that most studies focused on the general population or on health agencies, and only a small number of studies focused on ethnic minority groups, in accordance with existing reviews [12, 13]. Currently, there is a lack of knowledge on the modes of communication used to take into account the diversity of receivers in the field.

Second, no studies were identified that documented the effect on people’s self-protective behaviour related to health authorities’ use of social media platforms (e.g., Twitter, Facebook, Sina Weibo) and videos shared on YouTube. We identified studies on health authorities’ health risk communication on social media and outcomes pertaining to viewer engagement on YouTube and message retransmission on Twitter. However, previous studies have found that viewer engagement (number of views) is a poor predictor of usefulness, whereas the upload source and target audiences are good predictors of usefulness [98]. Future studies might benefit from studying the effectiveness of health authorities’ health risk communication on self-protective behaviour and include measures related to diverse upload sources, source credibility, and the effect of using different media types.

Third, the current review identified a limited number of studies of health authorities’ use of graphic and audio-visual means. The interdependence between graphic and audio visual means implies that modes presented together need to be interpreted with respect to one another [99]. More studies are needed on the effect of multimodal health risk communication in formal health risk communication. Health authorities’ videos were found to be credible with high quality, but they were not popular; they did not receive likes and comments, and they failed to engage the public. More studies are needed on how health authorities may include creative means in health risk communication with the public (e.g., evaluation of different *types* of narratives as well as creative choices in pandemic videos, evaluation of visual techniques for communicating data and numbers) without hampering their confidence and role as knowledge translators.

### Limitations

The present review is based on evidence retrieved from only two search databases. The search terms used were mainly Emtree terms and MeSH terms designed to capture articles in three areas: health and risk communication, media types and modes of communication, and pandemics. This may have resulted in the omission of relevant papers using different terms. This rapid scoping review explored the evidence and highlighted the key topics, research gaps and outcomes reported but did not compare studies or draw conclusions on causal relationships across studies and does not represent a concept analysis of the identified topics.

## Conclusion

The aim of this rapid scoping review was to synthesise the evidence regarding the different modes of communication used by health authorities in health risk communication with the public during a pandemic. This rapid scoping review identified studies in the interdisciplinary field of health risk communication mainly during the H1N1 pandemic and COVID-19 pandemic across three categories: communication channels, source credibility and how the message is communicated. A research gap identified in this review is the lack of high-quality RCT studies to study the effect of modes of pandemic health risk communication on behavioural outcomes. Content and engagement have been the major outcomes evaluated in social media, while effect studies on health authorities’ engagement in social media platforms (e.g., Twitter, YouTube) in pandemic health risk communication and the effect on self-protective behaviour are lacking. The evidence suggests that ethnic minority groups and migrant populations need personalised information and trusted spokespersons. However, there is a lack of studies on the modes of communication used to take into account the diversity of receivers in the field in pandemic health risk communication. More studies are needed across the fields of health risk communication and media studies (including visual communication, web design, video, and digital marketing) at a time when online digital communication is central to reaching the public.

## Supplementary Information

**Additional file 1.** Search strategy.

**Additional file 2.** Screening questions, eligibility criteria and data charting.

## Data Availability

The datasets used and/or analysed during the current study are available from the corresponding author on reasonable request.

## Abbreviations

“WHO”: World health organization
“COVID-19”: Coronavirus disease of 2019
“RCT”: Randomised controlled trial
