# Supplementary material for: Health authorities’ health risk communication with the public during pandemics: a rapid scoping review
Source: BMC Public Health. 2021 Jul 15;21:1401. doi: 10.1186/s12889-021-11468-3 (PMC8280576; doi:10.1186/s12889-021-11468-3)
Supplement: Supplementary file 1 — Additional file 1. Search strategy. [file 12889_2021_11468_MOESM1_ESM.docx]

**Additional file 1 Search strategy**

**Medline**

Search for: limit 11 to yr="2009 -Current"

Results: 948

Database: Ovid MEDLINE(R) ALL <1946 to October 27, 2020> Search Strategy:

--------------------------------------------------------------------------------

1 ((health or risk or mass or crisis or media or method* or mode*) adj2 (communicat* or literac*)).ti,ab,kf. (26148)

2 social media/ and (communicat* or literac*).ti,ab,kf. (1628)

3 ((online* or facebook* or youtube* or twitter or instagram or video* or internet* or tiktok or social media or

blog* or vlog* or online chat or online social network* or snapchat or snap chat) and (communicat* or literac*)).ti,ab,kf. (24834)

4 ((social adj2 (site* or platform* or mobile* or cell phone* or app or apps)) and (communicat* or

literac*)).ti,ab,kf. (897)

5 1 or 2 or 3 or 4 (46965)

6 Pandemics/ or Influenza A Virus, H1N1 Subtype/ or exp Coronaviridae/ or exp Coronavirus Infections/ (66446)

7 pandemic*.ti,ab,kf. (53936)

8 (("2009" or swine) adj2 (influenza* or flu* or pandemic*)).ti,ab,kf. (8664)

9 (coronavirinae or corona virus* or coronavirus* or pneumonia virus* or cov or covid19 or covid 19 or sars voc 2 or

sars2 or ncov 2019 or ncov or severe acute respiratory syndrome*).ti,ab,kf. (82530)

10 6 or 7 or 8 or 9 (124614)

11 5 and 10 (989)

12 limit 11 to yr="2009 -Current" (948)

**EMBASE**

Search for: limit 12 to yr="2009 -Current"

Results: 492

Database: Embase <1974 to 2020 October 27>

Search Strategy:

--------------------------------------------------------------------------------

1 ((health or risk or mass or crisis or media or method* or mode*) adj2 (communicat* or literac*)).ti,ab,kw. (35351)

2 social media/ and (communicat* or literac*).ti,ab,kw. (4020)

3 ((online* or facebook* or youtube* or twitter or instagram or video* or internet* or tiktok or social media or

blog* or vlog* or online chat or online social network* or snapchat or snap chat) and (communicat* or

literac*)).ti,ab,kw. (35365)

4 ((social adj2 (site* or platform* or mobile* or cell phone* or app or apps)) and (communicat* or

literac*)).ti,ab,kw. (1172)

5 1 or 2 or 3 or 4 (65347)

6 pandemic/ or pandemic influenza/ or 2009 H1N1 influenza/ or exp coronavirinae/ or Coronavirus infection/ (61827)

7 pandemic*.ti,ab,kw. (56664)

8 (("2009" or swine) adj2 (influenza* or flu* or pandemic*)).ti,ab,kw. (10906)

9 (coronavirinae or corona virus* or coronavirus* or pneumonia virus* or cov or covid19 or covid 19 or sars voc 2 or

sars2 or ncov 2019 or ncov or severe acute respiratory syndrome*).ti,ab,kw. (82029)

10 6 or 7 or 8 or 9 (125136)

11 5 and 10 (962)

12 limit 11 to embase (522)

13 limit 12 to yr="2009 -Current" (492)
