# Supplementary material for: Health authorities’ health risk communication with the public during pandemics: a rapid scoping review
Source: BMC Public Health. 2021 Jul 15;21:1401. doi: 10.1186/s12889-021-11468-3 (PMC8280576; doi:10.1186/s12889-021-11468-3)
Supplement: Supplementary file 2 — Additional file 2. Screening questions, eligibility criteria and data charting. [file 12889_2021_11468_MOESM2_ESM.docx]

**Additional file 2 Screening questions, eligibility criteria and data charting**

| **Eligibility criteria** |
| --- |
| **Inclusion criteria** |
| - Studies of modes of communication concerning pandemic information from health authorities to the public. Modes of communication in this paper included but were not limited to web-based information, social media, television, newspapers, video, texts, and narratives - Studies concerning health authorities pandemic communication including governments, official health experts, healthcare professionals as official spokespersons, health authority officials, health agencies, and official health bureaucrats, at the regional, national, or international level (i.e., the WHO). - Pandemics includes but not limited to: swine flu (H1N1) and Covid-19. - Are published during or after 2009 – this timeframe reflects the evidence generated following the last large scale pandemic (“Swine flu”), and also the need for evidence about communication modes to reflect the scale of technological change over the past decade. - Qualitative or quantitative empirical studies - Written in English |
| **Exclusion criteria** |
| - Health communication between individuals, such as a medical doctor and a patient or between healthcare professionals (e.g., digital educational methods, digital solutions). - Infectious diseases without a pandemic potential. - Published before 2009 - Commentaries, opinion pieces or other papers not reporting primary empirical research - Not written in English |

**Title/abstract screening**

Yes /maybe-> Full text screening. No-> exclude

- Is the study empirical?
- Does the study report on a pandemic context?
- Does the study report on public communication (as opposed to individual communication)?

**Full text screening for inclusion and exclusion**

- Is the study empirical?
- Does the study report on a pandemic context?
- Is the study peer- reviewed and published?
- Does the study reports on pandemic health risk communication from health authorities to the public?
- Does the study report on modes of communication used by health authorities?

**Data charting**

| **Reference** | **Origin** | **Context** | **Aim** | **Sample** | **Method** | **Types of outcomes and key topics** | **Modes of communication reported** | **Evidence related to mode of communication and health authorities** |
| --- | --- | --- | --- | --- | --- | --- | --- | --- |
|  |  |  |  |  |  |  |  |  |
